# Strategies for decarbonizing printed circuit board supply chain

**DOI:** 10.1016/j.isci.2026.115559

**Published:** 2026-04-08

**Authors:** Suraj Negi, Aishwarya Rani, Shu-Yuan Pan

**Affiliations:** 1 Department of Bioenvironmental Systems Engineering, College of Bioresources and Agriculture, National Taiwan University, Taipei 10617, Taiwan, ROC; 2 Agricultural Net-Zero Carbon Technology and Management Innovation Research Center, College of Bioresources and Agriculture, National Taiwan University, Taipei City 10617, Taiwan, ROC

**Keywords:** Environmental policy, Environmental science, Manufacturing

## Abstract

Printed circuit boards (PCBs) are indispensable to modern electronics and clean-energy technologies, yet their production relies on energy-intensive processes and fossil-based materials that contribute substantially to global greenhouse gas emissions. While numerous sustainability initiatives and technological innovations have been proposed across the PCB supply chain, their comparative effectiveness and scalability toward net-zero emissions remain unclear. This study synthesizes recent peer-reviewed life cycle assessments (LCA), ISO 14067-based product carbon footprint disclosures, industrial case studies, and policy initiatives to evaluate the environmental sustainability of the PCB supply chain from raw material extraction through manufacturing, distribution, and end-of-life management. By integrating boundary-qualified LCA evidence with industry practices, we identify dominant environmental hotspots associated with copper-intensive materials, electricity-demanding fabrication steps, and limited end-of-life circularity. Our analysis shows that many reported decarbonization measures are fragmented, regionally uneven, or constrained by data transparency, qualification requirements, and regulatory misalignment. To address these gaps, we present a unified synthesis framework comprising five decarbonization strategies: (1) renewable energy integration and energy-efficient manufacturing, (2) sustainable materials and design optimization, (3) circular economy strategies and targeted offsetting, (4) systematic LCA and product carbon footprint, and (5) harmonized industry standards and governance. Rather than treating these strategies as independent solutions, this study evaluates their interactions, feasibility constraints, and relative mitigation potential, providing a structured basis for advancing credible net-zero strategies in the global PCB supply chain.

## Graphical abstract

**Figure:**
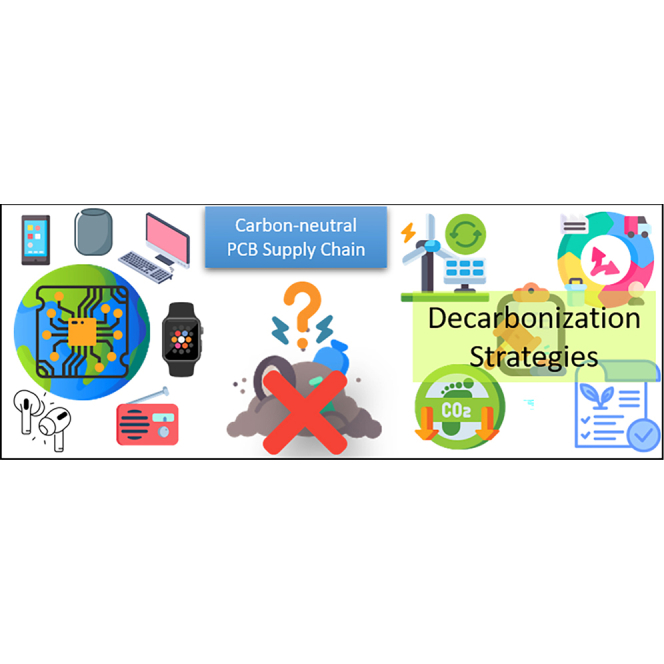

## Introduction

**Figure 1:**
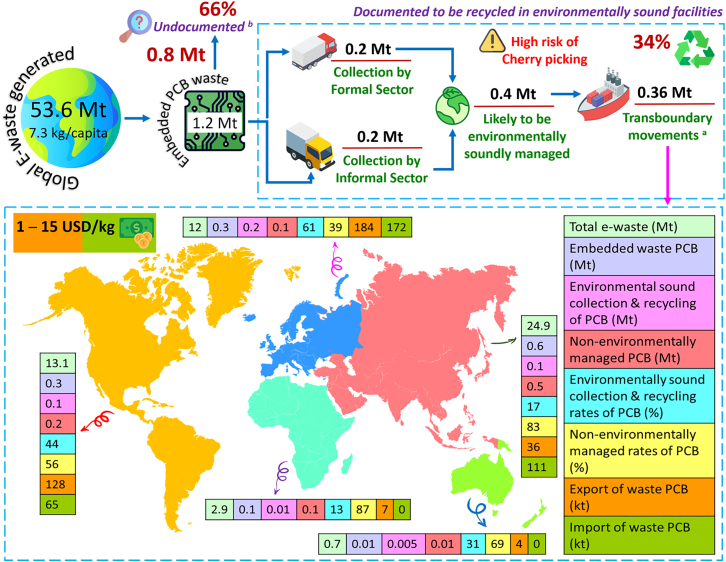
Global overview of PCB production and recycling Total maximum capacity of the waste PCB smelters = 0.5 Mt. Separated and recycled with backyard techniques (treated environmentally sound or dumped or recycled in a non-compliant or non-environmentally sound way). Out of all e-waste collected and treated, only 17% of it is done so formally. However, due to its high value, 34% of waste PCBs undergo proper, environmentally-friendly recycling, i.e., approximately half of these waste PCBs are collected and treated informally, while 66% of them are not officially documented to undergo safe and eco-friendly recycling procedures. The market rate of waste PCBs is 1–15 USD/kg. (Data extracted from C.P. Baldé.^8^) Credit: www.flaticon.com for the clipart.

In the last two decades, the electrical and electronic equipment (EEE) industry has expanded rapidly, with the amount of EEE placed on the global market increasing from 62 billion kg in 2010 to 96 billion kg in 2022 and projected to reach 120 billion kg by 2030.^1^ Printed circuit boards (PCBs) are foundational to this growth, functioning as the structural and functional backbone of virtually all electronic products, including consumer devices, vehicles, industrial systems, medical technologies, and clean-energy infrastructure. The global PCB market is valued at approximately US$70−80 billion in 2025^2^ and is expected to exceed US$100 billion by 2030–2032,^3^^,^^4^ driven by trends such as electric vehicles,^5^ 5G, the internet of things (IoT), and healthcare electronics. Production is currently concentrated in the Asia-Pacific region, particularly China, although geopolitical pressures are driving partial supply-chain diversification toward emerging hubs such as Thailand.^6^ Key players include Taiwanese, Japanese, and Chinese firms that focus on high-density interconnect (HDI) and flexible PCBs for advanced devices.^7^ Figure 1 shows a global overview of PCB production, waste generation, collection, recycling, and transboundary movements, adapted from the Global Transboundary E-waste Flows Monitor 2022.^8^ Although the underlying dataset is reported at the EEE level, it represents the most comprehensive global source available for tracing PCB-containing waste flows, as PCBs are embedded in virtually all electronic products and constitute a high-value, high-impact fraction of e-waste streams. The figure should therefore be interpreted as a best-available system-level representation of PCB flows rather than a complete, PCB-exclusive mass balance.

As electronics demand rises, so does the sector’s climate footprint. Recent synthesis studies estimate that electronics account for more than 4% of global greenhouse gas (GHG) emissions, with upstream supply-chain activities (scope 3), including component and PCB manufacturing, contributing to the majority of emissions.^9^ This shifts the decarbonization challenge away from final assembly alone and toward earlier, less transparent stages of the value chain. In parallel, global e-waste generation rose from 34 billion kg in 2010 to 62 billion kg in 2022 and is projected to reach 82 billion kg by 2030.^1^ Despite incremental improvements in formal collection and recycling, growth in e-waste continues to outpace recovery capacity, particularly in lower-income regions. PCBs play a disproportionate role in both the carbon and material intensity of electronics. Conventional PCB manufacturing relies on carbon-intensive metals (e.g., copper and gold), fossil-derived laminates (e.g., epoxy resins and glass fiber), and energy- and chemistry-intensive processes such as etching, electroplating, drilling, and lamination. PCBs constitute only 3%–6% of e-waste by mass yet contain approximately 7% of the world’s gold, making them both a valuable secondary resource and a significant source of environmental and health risk when improperly managed.^10^^,^^11^ Informal recycling and disposal practices, particularly in parts of Africa and Asia, expose workers and surrounding communities to toxic emissions, heavy metals, and persistent organic pollutants.^12^^,^^13^ These characteristics make PCBs a particularly stubborn bottleneck for net-zero electronics: GHG emissions are embedded across scope 1–3 (here used as a conceptual mapping to ISO 14064/14067 categories 1–6),^14^^,^^15^ materials are tightly integrated into metal-polymer composites, and end-of-life separation remains technically and economically challenging.^16^ More details on the waste PCBs can be found in the Table S1. It is also interesting to analyze data from Figure S1, which shows that the per capita waste PCB production is highest in Europe and lowest in Africa. A similar trend is observed for formal PCB waste collection and recycling, with Europe leading and Africa lagging behind. Detailed figures on per capita PCB waste generation, collection, as well as import and export, are provided in Table S2.

Although “green electronics” innovation is accelerating, the sustainability outcomes remain uneven. Approaches such as printed and additive electronics promise reductions in material use and subtractive processing. However, evidence suggests that incremental optimization of conventional PCB technologies may be insufficient to achieve deep decarbonization at scale.^17^ Technical feasibility, application constraints, data opacity, and end-of-life trade-offs continue to limit the real-world impact of many proposed solutions. At the same time, international policy frameworks, including the Basel Convention, the European Union’s Waste Electrical and Electronic Equipment (WEEE) Directive; registration, evaluation, authorization and restriction of chemicals (REACH); and restriction of hazardous substances (RoHS),^18^^,^^19^^,^^20^ have improved hazardous-waste governance but have not yet delivered harmonized, supply-chain-wide decarbonized pathways for PCBs. Existing circular economy and sustainability research in the electronics sector is expanding rapidly, yet recent synthesis work finds it remains fragmented, often qualitative/exploratory, and lacks comprehensive frameworks that reliably guide implementation across complex electronics value chains.^21^ In parallel, PCB-focused literature frequently concentrates on individual slices of the problem (e.g., manufacturing steps, material substitutions, or recycling methods) rather than integrating raw-material sourcing → fabrication/assembly → logistics → end-of-life into a unified decarbonization view tied to measurable emissions hotspots and governance mechanisms. Importantly, reported decarbonization claims for PCB-related interventions vary widely in magnitude, system boundary, and verification level, ranging from incremental efficiency gains to large, case-specific reductions, making it difficult to assess their relative effectiveness without a unified, quantitative evaluation framework.

Against this backdrop, this review synthesizes decarbonization strategies across the full PCB supply chain to evaluate whether existing measures plausibly support net-zero transitions. Specifically, it (1) identifies life cycle environmental and carbon-emission hotspots across PCB materials, manufacturing, distribution, and end-of-life stages; (2) consolidates reported carbon-footprint and life cycle assessment (LCA) evidence to support cross-study comparison; and (3) proposes five interconnected decarbonization strategies, distinguished by their dominant emission scopes, life cycle hotspots, and feasibility constraints, spanning renewable energy and energy efficiency, sustainable materials and design optimization, circular economy strategies, systematic LCA, and harmonized standards and regulation, to translate circular-economy principles into implementable, supply-chain-level action.

## Methods

We conducted a problem-oriented narrative review^22^ with scoping elements to synthesize decarbonization options across the PCB supply chain. The review was guided by two questions: (1) whether the PCB supply chain is environmentally sustainable, and (2) whether current practices are sufficient to meet carbon-neutral or net-zero targets. Evidence was organized by life cycle stage (raw material sourcing, fabrication, assembly, logistics, and end-of-life) and synthesized into five strategies spanning energy supply and efficiency, sustainable materials and design, circularity strategies (including recycling and recovery), LCA, and standards/regulation.

To strengthen transparency and update recent coverage during revision, we used targeted keyword searches in Scopus (document types: Article/Review; Language: English; subject areas: Engineering, Environmental Science, Energy, Materials Science) across 2010–2025, with a focused update window of 2023–2025. Six query families captured PCB LCA/carbon footprint evidence, manufacturing decarbonization, and process hotspots, waste PCB recycling impacts, sustainable/biodegradable substrates, circular economy/design-for-recycling strategies, and additive/printed electronics routes (full query strings and per-family record counts in Supplementary Sections (S1−S5). Records were screened at the title/abstract level for relevance to PCBs and supply-chain decarbonization, including exclusion of results where “PCB” referred to polychlorinated biphenyls. Rather than aggregating values, evidence was synthesized by comparing reported magnitudes, system boundaries, and identified hotspots across studies, enabling a feasibility- and boundary-aware comparison of decarbonization strategies. This review does not claim exhaustive capture of all PCB sustainability literature; selection reflects relevance to the study questions and the availability of comparable evidence. Studies were included if they provided quantitative or semi-quantitative evidence relevant to PCB decarbonization, evaluated technological or organizational interventions along the PCB supply chain, or addressed governance mechanisms affecting PCB-related emissions. Studies focusing on unrelated electronics components or non-PCB materials were excluded.

## PCB manufacturing and supply chain

### Process overview

**Figure 2:**
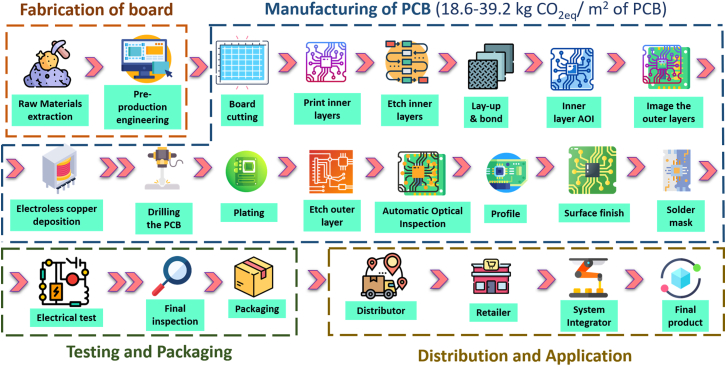
Supply chain of PCB including fabrication of board, manufacturing of PCB, testing and packaging, distribution and application, and end-of-life (Carbon footprint data were extracted from Yung et al.^23^) Credit: www.flaticon.com for the clipart.

The PCB supply chain (Figure 2) spans raw-material sourcing, board fabrication, PCB manufacturing, testing/packaging, distribution, and use in electronic products, and end-of-life management.^24^ PCBs are multilayer metal-polymer composites in which conductive copper layers are integrated with polymeric laminates (e.g., epoxy/glass-fiber systems such as flame-retardant (FR-4)^25^) and protected by solder masks and surface finishes. Because these materials and processes are tightly coupled, environmental burdens arise from both upstream materials (e.g., metals, laminates, and chemicals) and downstream fabrication operations (e.g., energy-, water-, and chemical-intensive processing).

In Figure 2, upstream impacts originate mainly from copper-intensive materials, laminate/resin production, and the supply of process chemicals. These inputs feed a largely subtractive fabrication sequence (imaging → etching→ lamination → drilling → metallization/plating → surface finishing), followed by testing/packaging and distribution to downstream product assembly. End-of-life pathways (formal recycling, informal recycling, export, or disposal) determine whether metals are recovered and whether the non-metallic fraction becomes a persistent waste burden, making design-for-recycling and collection infrastructure central to realizing life cycle emission reductions.

Conventional PCB manufacturing is largely subtractive and proceeds through repeated cycles of pattern transfer and copper removal/addition. Inner layers are patterned using photoresist imaging,^26^ followed by etching of unwanted copper. Etching is particularly material- and waste-intensive: prior studies report that a substantial fraction of copper originally present on the panel (often cited as ∼50%–70%^27^) can be dissolved during etching, motivating copper recovery from spent etchants and improved etchant management. After inner-layer fabrication, multilayer boards are formed by lamination/pressing of stacked cores and prepregs; lamination materials and resin systems strongly affect both footprint and end-of-life separability. Drilling (mechanical and/or laser) creates vias and component holes, followed by desmear and metallization steps. Drilling can be both energy-consuming and costly, and optimization of machining parameters has been shown to reduce energy use and manufacturing cost.^28^

Metallization and plating then build conductive thickness and interlayer connectivity (e.g., plated through-holes and blind vias). Process innovations in plating (e.g., surfactants and supercritical CO_2_-assisted electroless plating routes) aim to improve deposition quality and corrosion resistance,^29^ but also influence bath chemistries, energy demand, and wastewater loads. Final stages typically include solder mask application, surface finishing (e.g., hot air solder leveling, electroless nickel immersion gold plating, and organic solderability preservative), marking/legend printing, profiling, and cleaning.^30^^,^^31^^,^^32^ Across these steps, large volumes of rinse water and diverse chemical baths generate metal-bearing wastewater, spent solutions, and treatment sludges, making onsite wastewater treatment and waste handling central to environmental performance.^33^^,^^34^^,^^35^

Testing and inspection (e.g., automated optical/visual inspection and electrical tests such as in-circuit and functional testing) are essential for reliability and safety. From a sustainability perspective, their main relevance is indirect: yield loss, rework, and scrap can substantially increase per-board energy and chemical burdens, so defect prevention and yield improvement function as emissions- and waste-reduction levers even when testing itself is not a dominant hotspot.

### Environmental impact

PCB production generates multiple environmental stressors, including energy-related emissions, hazardous chemical releases, metal-containing effluents, and solid wastes. During fabrication, materials such as copper, aluminum, and lead can release harmful substances (including toxic metals) if improperly handled, while process chemicals and additives (e.g., brominated flame retardants and related compounds) introduce toxicity concerns across manufacturing and end-of-life stages.^33^^,^^35^^,^^36^ Consistent with this, the PCB manufacturing process is associated with substantial waste and wastewater streams, including hazardous spent etchants/strippers, metal-bearing sludges, and contaminated solid residues. Table 1 summarizes representative waste quantities generated per square meter of multilayer PCB production.

**Table 1:** Amount of waste from the multilayer printed circuit^37^ board manufacturing process

Characterization	Waste	Mass or volume per m^2^ of PCB
Hazardous	waste board	0.01–0.3 kg/m^2^
	edge trim	0.1–1.0 kg/m^2^
	hole drilling dust	0.005–0.2 kg/m^2^
	tin/lead dross	0.01–0.05 kg/m^2^
	wastewater treatment slurry	0.02–3.0 kg/m^2^
	acidic etching solution	1.5–3.5 L/m^2^
	basic etching solution	1.8–3.2 L/m^2^
	rack stripping solution	0.2–0.5 L/m^2^
	tin/lead stripping solution	0.2–0.6 L/m^2^
	sweller solution	0.05–0.1 L/m^2^
	flux solution	0.05–0.1 L/m^2^
	microetching solution	1.0–2.5 L/m^2^
	Pth copper solution	0.2–0.5 L/m^2^
Non-hazardous	copper powder	0.001–0.01 kg/m^2^
	copper foil	0.01–0.05 kg/m^2^
	alumina plate	0.05–0.1 kg/m^2^
	film	0.1–0.4 kg/m^2^
	drill backing board	0.02–0.05 kg/m^2^
	paper (packaging)	0.02–0.05 kg/m^2^
	wood	0.02–0.05 kg/m^2^
	container	0.02–0.05 kg/m^2^
	paper (processing)	–
	ink film	0.01–0.1 kg/m^2^
	garbage	0.05–0.2 kg/m^2^

This table reports representative waste-generation ranges for multilayer PCB manufacturing based on a Taiwanese subtractive-process case study. Absolute quantities may vary with board design, layer count, process technology, and regional regulations. However, the table is retained to illustrate the diversity and magnitude of waste streams relevant to PCB environmental management.

#### Carbon emissions and comparability challenges

Carbon emissions are increasingly emphasized because they connect PCB manufacturing directly to net-zero strategies. Despite the importance, very few studies have performed the LCA of PCBs and reported the carbon footprint. For example, the carbon emissions for raw material extraction (Figure 2) are ∼122 kg CO_2_eq per m^2^ of PCB, followed by manufacturing, i.e., 160.55 kg CO_2_eq per m^2^ of PCB.^23^ A widely cited sector-level estimate suggests the PCB industry contributes on the order of 66 million tonnes CO_2_eq per year (∼0.13% of global emissions).^38^ However, this figure is based on industry-reported scaling assumptions and should be interpreted as an order-of-magnitude indication rather than a precise inventory. At the facility and product level, reported PCB carbon footprints differ markedly because studies use different functional units (e.g., kg CO_2_eq per m^2^, per board, per mass), system boundaries (gate-to-gate, cradle-to-gate, and cradle-to-grave), board complexity (layer count, materials, finishes), yield assumptions, and electricity-grid emission factors.^39^^,^^40^^,^^41^^,^^42^^,^^43^ To prevent inappropriate “apples-to-oranges” comparisons, Table 2 consolidates peer-reviewed LCA/carbon-footprint values and product carbon footprint disclosures (in accordance with ISO 14067), explicitly reporting functional units and stated system boundaries.

**Table 2:** Reported carbon footprints for printed circuit board (PCB) production across peer-reviewed life cycle assessments and ISO 14067-based product carbon footprint disclosures

Source	Object	System boundary	Functional unit	Result reported	What it implies for hotspots
PCB LCA^39^	single-layer PCB	excludes transport/used/EoL; includes board fabrication + manufacturing steps	1 m^2^ PCB	18.6 kg CO_2_eq/m^2^	manufacturing stage ∼38% of total GWP; within manufacturing, etching ∼33%, solder mark ∼20%, coatings ∼19%, printing ∼13%
Dynamic Electronics ISO-style PCF^40^	10-layer PCB	cradle-to-gate (explicit)	1 ft^2^ PCB	16.6347 kg CO_2_eq/ft^2^ (=∼179 kg CO_2_eq/m^2^)	manufacturing = 58.17% of total; within manufacturing, outsourced electricity = 92.81%
Kinwong ISO 14067 certificate^41^	rigid 6-layer PCB	raw materials + transport + core production + product transport (as stated)	1 piece	37.75 kg CO_2_eq/pieces	useful real-world benchmark, but not area-normalized (limits comparability).
Ochoa et al.^42^	PWBs across designs	cradle-to-gate	per board/per m^2^ (varies in paper)	∼0.6–10 kg CO_2_eq/board; dominated by manufacturing energy + laminate (∼80%)	impacts vary with design parameters beyond area + layer count; electricity EF is a top uncertainty driver; databases can be ∼1.5–2.6× higher than facility-based results.
Fairphone 5 LCA^43^	primary PCBA	product-system LCA (phone modules)	per module	Primary PCBA 17.8 kg CO_2_eq; ICs 16.2 kg CO_2_eq; PCB 1.47 kg CO_2_eq	context: for “electronics net-zero”, PCB matters, but the IC supply chain often dominates assembly footprints.

Emission categories are defined according to ISO 14064/14067 (Categories 1–6). References to Scope 1–3 in the text reflect a conceptual mapping only and are not used for accounting or aggregation. Here, PCB: printed circuit board, LCA: life cycle assessment, ISO: International Organization for Standardization, PCF: Product Carbon Footprint, PWB: Printed Wiring Board, PCBA: printed circuit board assembly, EoL: End of Life, IC: Integrated Circuits, GWP: Global Warming Footprint, EF: Emission Factor.

Values are reported using the original functional unit and system boundary (e.g., per m^2^, per ft^2^, or per piece; cradle-to-gate vs. partial boundaries), and are therefore intended for benchmarking and hotspot identification rather than direct like-for-like comparison.

#### Hotspots indicated by LCAs and product carbon-footprint disclosures

Despite heterogeneity, the quantitative literature converges on several robust hotspot patterns. Etching and wet processing repeatedly emerge as major contributors across impact categories. In a streamlined LCA of a PCB manufacturing facility, PCB production contributed ∼89% of freshwater aquatic ecotoxicity potential and ∼73% of ozone depletion potential, with etching identified as the primary hotspot in nearly all impact categories (except eutrophication).^39^ Within that same case, etching contributed ∼33% of the manufacturing-stage global warming potential, with additional contributions from solder mask and protective coating operations.^39^ These results are consistent with the broader understanding that wet processing drives both chemical burdens and downstream treatment/sludge impacts, particularly when copper-bearing residues are incinerated or otherwise managed in ways that intensify toxicity-related indicators.

Electricity and laminate materials dominate cradle-to-gate climate impacts when assessed with primary/facility data and representative designs. Design- and facility-informed work on printed wiring boards (including HDI designs) reports cradle-to-gate impacts that vary strongly by design and application (e.g., handheld devices vs. notebooks/desktops), with global warming potential dominated by manufacturing energy and laminate materials (together ∼80%) and with additional sensitivity to design parameters beyond layer count and board area.^42^ This is a critical methodological point for this review: board “complexity” (and not area alone) can shift both total impact and hotspot ranking.

ISO 14067-based product carbon footprints provide real-world benchmarks that emphasize electricity dominance in manufacturing.^40^ A cradle-to-gate product carbon footprint report for a 10-layer PCB (declared unit: 1 square foot) reports a total footprint of ∼16.635 kg CO_2_eq per declared unit, with the manufacturing stage contributing ∼58% of the total footprint.^40^ Within that manufacturing-stage footprint, outsourced electricity contributed ∼93%, indicating that decarbonizing electricity supply and improving energy efficiency are decisive levers for many modern multilayer boards. A separate ISO 14067 verification opinion for a rigid six-layer PCB reports a cradle-to-gate footprint of ∼37.75 kg CO_2_eq per piece (including raw material acquisition and transport, core production, and product transportation), further illustrating that declared units and product specifications must be stated explicitly when using product carbon footprints for benchmarking.^41^ These disclosures complement academic LCAs by offering boundary-defined, audit-verified snapshots of specific board types and facilities.

#### Linking PCB-level evidence to product-level emissions

PCBs ultimately contribute to the embodied footprint of electronic products through both the board itself and the components assembled onto it. Product-level LCAs indicate that production often dominates total life cycle impacts for electronics, and within production, PCB assemblies (PCBAs) can be major contributors depending on device architecture and component mix.^43^ For example, in the Fairphone 5 LCA, the primary PCBA is a dominant module contributor to production impacts, while the majority of primary-PCBA impacts are attributed to integrated circuits, with most of the remaining impacts attributed to the PCB itself.^43^ This reinforces a key framing for the strategies developed later in the study: PCB decarbonization must be treated as a supply-chain problem that includes electricity and process chemistry at fabrication sites, upstream laminate/metal supply, and, at the system level, how PCB design and assembly interact with component choice and product lifetime.

### Implications for decarbonization priorities

Taken together, PCB manufacturing and supply chain establishes three actionable conclusions that motivate the decarbonization strategies developed in later sections: (1) PCB manufacturing burdens are driven by a combination of upstream materials and downstream wet processing; (2) hotspot patterns are consistent (e.g., electricity, laminates/materials, and etching/plating chemistry plus wastewater/sludge management), but their relative importance depends on board complexity, yields, and local energy mixes; and (3) meaningful comparison of PCB carbon footprints requires explicit reporting of functional unit and boundary (Table 2), and should rely on boundary-qualified ranges rather than a single “typical” value.

## Global initiatives for sustainable PCBs

There are a number of opportunities for improving the environmental performance or sustainability of the PCB supply chain, including efficient product design, sustainable sourcing of materials, improved manufacturing processes, efficient waste management, and improved recycling. The product design can be improved by implementing the Design for the Environment tool^44^^,^^45^^,^^46^ and applying green chemistry principle 10 (including design for degradation),^47^ and it should support modular devices, allowing for easy replacement of individual components, and designing devices. Another way to decarbonize the PCB supply chain is to source metals from responsible mines and use environmentally friendly materials such as recycled plastics and resins. In the manufacturing stage, the application of green chemistry principles^48^ for reducing the use of toxic chemicals, increasing the use of environmentally friendly technologies, and reducing waste can improve the sustainability of manufacturing processes. Moreover, enhancing e-waste collection and recycling rates can lead to greater efficiency in PCB recycling processes.

**Figure 3:**
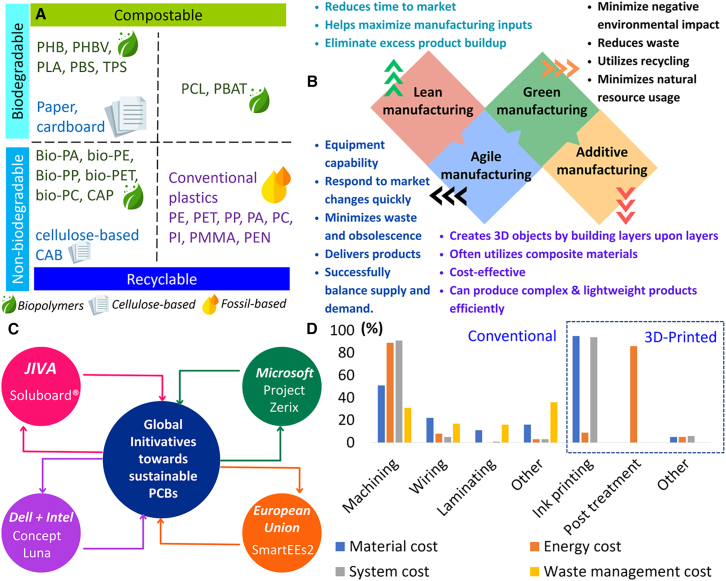
Global initiatives and regulatory pathways to decarbonize the PCB supply chain (A) Categorization of different plastic materials,^49^ (B) PCB manufacturing strategies tailored toward sustainability,^50^ (C) Bigger global initiatives toward sustainable PCBs, and (D) The material, energy, system, and waste management costs in conventionally manufactured PCB vs. 3D printed PCBs.^51^ Credit: www.flaticon.com for the clipart.

To reduce case-listing” and improve comparability across initiatives, the following examples are discussed using a consistent interpretation lens: (1) the life cycle lever targeted (materials/design, manufacturing energy/process, logistics, end-of-life), (2) the emissions scopes most affected (scope 1–2 vs. scope 3), (3) maturity/readiness (prototype, pilot, commercial adoption), and (4) key feasibility constraints (qualification/reliability, cost, supply-chain traceability, and end-of-life infrastructure). Reported “% reductions” (carbon, water, copper, and plastics) are treated as case-specific unless the functional unit and system boundary are clear, because outcomes can shift with board complexity, yield/scrap, and electricity mix (see environmental impact; Table 2). Regional context also matters: many disclosure and procurement pressures originate in Europe/North America, while much PCB fabrication capacity sits in Asia-Pacific; therefore, supply-chain initiatives scale fastest when they translate into supplier requirements and verified reporting across upstream fabrication and material suppliers (Figure 3).

### Companies’ case studies

In this section, we discussed the sustainable initiatives and regulatory pathways adopted by companies to decarbonize the PCB supply chain, as shown in Figure 3. JIVA^52^ built Soluboard as a fully bio-degradable green alternative to the non-sustainable material within the conventional PCB, i.e., glass-reinforced epoxy resin laminate (FR-4).^53^ The primary material in Soluboard is a natural fiber that serves as an alternative to traditional glass-based fiber, with its multilayer bio-composite design meeting the mechanical and electrical standards of the electronics industry. Owing to its organic composition, the board delaminates when placed in hot water, enabling the organic fibers to be composted and allowing for the recovery and recycling of 90% of the electrical components.^54^ Soluboard PCB (7.1 kg CO_2_/m^2^; 1.35 g/cm^3^) can reduce up to 60% of the carbon footprint and 620 g/m^2^ of plastic savings^54^ if replaced by standard FR-4 PCB (17.7 kg CO_2_/m^2^; 2 g/cm^3^). A recent study by the University of Washington^55^ used a Soluboard circuit board to redesign an eco-friendly mouse and compared it with an FR-4 circuit board-based mouse. Research has shown that using Soluboard in place of conventional materials can lower carbon emissions by ∼60%. Infineon Technologies AG, Germany’s largest semiconductor producer, has developed water-soluble PCB prototypes based on the Soluboard platform.^56^ This water-based recycling method can improve the recovery rate of valuable metals. Additional information on organic-based PCBs is provided in the supplementary Table S3. Feasibility and constraints: Soluboard primarily targets scope 3 material emissions (laminate substitution) and end-of-life separability. However, scaling depends on qualification against mainstream reliability requirements (e.g., thermal cycling, moisture sensitivity, and solder reflow compatibility) and on whether collection/take-back routes enable the intended delamination recovery pathway rather than generic mixed e-waste treatment.

Similarly, the Tokyo-based startup Elephantech Inc. has introduced an eco-friendly PCB, known as P-Flex, manufactured via metal inkjet printing. This approach involves fewer production steps, reducing both environmental impact and costs.^57^ The process creates the desired copper patterns by printing metal onto film and growing it, eliminating the need for copper foil, copper-clad laminate fabrication, and etching processes that dissolve and discard excess copper. Elephantech^57^ claimed a decrease in copper consumption by ∼70%, carbon footprint by ∼75%, and water footprint by ∼95%. Eizo Corporation has adopted the P-Flex for the FlexScan EV3895 curved monitor and its capacitive touch switches. Also, Fukuda Co., Ltd. employed the P-Flex in the pressure sensor module in the air leak test system. Feasibility and constraints: P-Flex targets manufacturing-stage hotspots by reducing subtractive etching and associated copper-bearing wastes (consistent with environmental impact hotspot patterns). Manufacturer-reported reductions should be interpreted as product- and boundary-dependent unless disclosed with transparent inventories and third-party verification. Wider adoption will depend on throughput, multilayer capability, defect/yield performance, and compatibility with high-density interconnect requirements.

Winbond electronics announced that its flash memory products are now compatible with the low-temperature soldering (LTS) process, lowering the Surface Mount Technology temperature in lead-free production from 260 °C to 190 °C. Adopting the LTS process can significantly cut both carbon emissions and related costs.^58^ Feasibility and constraints: LTS mainly targets scope 2 emissions by reducing reflow energy demand and may also reduce thermal-stress-related defects (yield/rework). Net benefit depends on solder alloy availability, reliability under thermal cycling, and whether upstream impacts from specialty materials offset downstream energy savings.

Apple, one of the world’s largest electronics manufacturers, has also pledged to reduce its carbon footprint and achieve carbon neutrality across its operations, manufacturing supply chain (including suppliers), and entire product life cycle by 2030.^59^ Concerning the PCB board, Apple plans to reduce the copper needed to build key components by using foils that rely less on copper in PCBs and using 100% recycled tin in the solder of multiple PCBs.^60^ Apple built the PCB for the iPhone 12 with 50% less gold plating than the iPhone 11 and plans to use 100% certified recycled gold on the plating of circuit boards for the iPhone by 2025.^60^ Feasibility and constraints: These measures mainly target scope 3 material intensity (metals). The primary constraint is supply-chain traceability and scalable availability of certified recycled content, rather than technical feasibility; credible impact requires chain-of-custody systems and supplier verification rather than “paper decarbonization.”

Microsoft launched project ZERIX, i.e., zero environmental impact materials for a sustainable future, which aims to achieve net-zero embodied carbon and net-zero waste by exploring new biological and chemical circularity pathways. Microsoft developed more sustainable PCB substrate materials from renewable materials in collaboration with the world institute for sustainable development of materials (WISDOM) research group, University of California, Irvine. WISDOM group is establishing an interpretive framework for various end-of-life pathways for e-waste management with a focus on PCBs to inform best practices and decisions to invest in more environmentally sustainable PCBs. A team from the University of Washington and Microsoft published an article about sustainable mouse design by replacing the FR-4 circuit board with a Soluboard circuit board.^55^ Feasibility and constraints: This initiative is stronger than “materials-only” approaches because it explicitly links substrate changes to end-of-life pathway feasibility. Translating pilots into supply-chain-level reductions requires measurable supplier data (product carbon footprint/LCA inventories) and procurement criteria that push adoption beyond demonstration products.

Dell Technologies Inc. built a laptop with bio-based components in regard to their GHG reduction initiatives to cut emissions in half by 2030.^61^ To reduce the carbon emissions from PCBs, Dell launched the concept Luna, in which they introduced bio-based PCBs made of flax fiber and used a water-soluble glue that replaced plastic laminates found in conventional circuit boards. The concept of Luna increases the recycling rate of metals and electrical components at the end of life. Intel has also partnered with Dell Technologies Inc. in the Concept Luna and pledged to achieve net-zero GHG emissions globally by 2040.^62^ Feasibility and constraints: Concept Luna emphasizes design-for-disassembly and design-for-recycling, targeting end-of-life separability as the enabling condition for higher recovery yields and reduced virgin-material demand. Realized benefit depends on collection, controlled dismantling, and recycling capacity; without take-back systems, design improvements may not translate into higher actual recovery.

Similarly, Taiwan Printed Circuit Association^63^ has committed to cutting the PCB industry’s emissions in Taiwan by 30% by 2030. Its sustainable carbon reduction strategy focuses on two main initiatives: (1) introducing low-carbon equipment for key process hotspots, particularly electroplating and factory facilities, and (2) establishing a low-carbon materials R&D platform to develop environmentally friendly materials for copper-clad laminates, resins, and dielectric components. Feasibility and constraints: this case is supply-chain-relevant because it targets the manufacturing geography where much PCB fabrication occurs. Its effectiveness depends on whether “low-carbon equipment” translates into measurable scope 2 reductions (energy efficiency, electrification, and renewable electricity procurement) and whether material R&D is coupled to standards/qualification and adoption mechanisms rather than voluntary targets.

Across these company cases, the dominant levers cluster into (1) material substitution and recycled-content strategies (scope 3), (2) manufacturing process redesign and energy measures (scope 2, plus chemistry/waste co-benefits), and (3) design-for-disassembly that enables higher recovery at end-of-life. In practice, scaling constraints are usually qualification/yield economics and end-of-life infrastructure, not a lack of conceptual solutions.

### R&D initiatives

The successful commercial, sustainable solutions for PCBs were once lab-scale R&D initiatives. This section highlights several selective R&D initiatives toward sustainable or carbon-neutral PCBs. The greener PCBs need sustainable designs and manufacturing. Sustainable manufacturing ensures minimal impact on the environment by minimally consuming resources and saving them for future generations as well. Deng et al.^64^ conducted a comparative LCA and reported better performance of natural fiber alternatives and bio-based material than epoxy resins and glass fibers. In another study, epoxy resin and glass fiber were substituted with epoxidized linseed oil and flax fibers, respectively, while still meeting most of the requirement categories specified in IPC-4101A/20.^65^ Other research has explored biopolymers such as polyhydroxybutyrate, cellulose acetate, and two different copolymers, polylactic acid (PLA) with thermoplastic polyester elastomer (PLA+TPC) as potential materials for flexible PCBs.^66^ Feasibility and constraints: These approaches primarily reduce scope 3 impacts embedded in laminate systems, but must demonstrate IPC-relevant reliability and manufacturability in realistic multilayer builds (not only material coupons). “Qualified substitution” (replacing specific layers/adhesives in suitable applications) is more scalable than assuming immediate drop-in replacement across all PCB classes.

Danninger et al.^67^ demonstrated the use of a fungal mycelium “skin” derived from the saprophytic fungus, *Ganoderma lucidum*, as a biodegradable substrate for PCB production. This material supports physical vapor deposition and laser patterning for electronic traces, achieving conductivities up to (9.75 ± 1.(44) × 10^4^ S cm^−1^, withstanding more than 2,000 bending cycles, and tolerating multiple folds with only moderate increases in resistance. Huang et al.^68^ and Rogers and Huang^69^ developed a transient PCB using two separately fabricated sodium carboxymethylcellulose (Na-CMC) substrates bonded with poly(ethylene oxide) (PEO), featuring electrical interconnects made from transient metals such as magnesium, tungsten, or zinc. Both Na-CMC and PEO are water-soluble and biocompatible. Also, bioabsorbable materials such as poly (lactide-co-glycolide), poly(glycolide), and PLA could be used as an alternative for PCB substrate. Cellulosic PCB with laser-induced graphene as conductive material using a screen printing technique is an integration of a sustainable and cost-effective substrate, conductive circuits, and processes for PCB manufacturing.^70^ Feasibility and constraints: transient/bioabsorbable PCB concepts are most immediately suitable for short-lifetime or controlled-degradation applications (e.g., biomedical and disposable sensors). For mainstream electronics, “degradability” must be decoupled from premature failure; therefore, these approaches are likely to scale first in niche products unless paired with robust encapsulation strategies and clearly defined end-of-life triggers.

Krishna and Srikanth^71^ compared the environmental impact of additive (fused deposition modeling) and subtractive (computer numerical control machining) manufacturing for PCBs. Their findings showed that, in terms of resource consumption, additive manufacturing has nearly 30% less environmental impact than subtractive methods. Lean manufacturing approaches, such as Just-in-Time, Kanban, Single Minute Exchange of Die, and Value Stream Mapping, can also help streamline operations and facilitate the shift from batch to continuous assembly production.^72^ Jeswiet and Kara^73^ introduced a straightforward “carbon emission signature” method to quantify GHG emissions during product manufacturing. Rajemi et al.^74^ developed a model and methodology to optimize the energy footprint of machined products. Their approach incorporated miniaturization through molded interconnect devices, a rapidly growing design optimization technique that enables high functional integration, reduces part counts, improves design flexibility, and delivers significant sustainability advantages. Feasibility and constraints: Additive and lean approaches can reduce wet-chemistry burdens, scrap, and rework (aligning with environmental impact hotspots), but net decarbonization depends on throughput, defect/yield performance, and electricity intensity of printing/curing steps. Operational improvements often deliver “intensity” gains; aligning with net-zero requires coupling them to absolute emissions targets and verified accounting.

In the context of Industry 4.0, the entire product life cycle value chain will become individualized, demand-driven, intelligently organized, and cognitively managed through the analysis of design, production, sourcing, and inventory data. Deep learning models can provide highly accurate forecasts regarding the timing and characteristics of incoming orders over a given planning horizon. Building on this concept, Leng et al.^75^ developed a hybrid real-time PCB order acceptance decision model that integrates deep learning with reinforcement learning in a loosely coupled framework. This approach enables the prediction of key cleaner production indicators, such as cost, production time, and carbon consumption, for PCB orders in advance, allowing manufacturers to strike an optimal balance between profitability, carbon footprint, and customer priority. Feasibility and constraints: Digital optimization reduces emissions indirectly via better planning and lower scrap, but it does not guarantee net-zero alignment unless linked to verified emissions inventories and constraints that limit absolute emissions (not only carbon intensity per order).

PCB recycling is a critical issue, as only a portion of waste PCBs is recycled. The recycling consists of three phases, i.e., pretreatment (disassembling reusable and hazardous components through separation and shredding), physical recycling (mechanical methods such as shape-based separation, magnetic separation, electrical conductivity-based, density-based, and electrostatic separation), and chemical recycling (methods like gasification, pyrolysis, depolymerization, and hydrogenolic degradation).^72^ Several environmentally friendly or low-carbon recycling attempts for PCB have been performed in the literature. For example, Chen et al.^76^ applied small-molecule-assisted dissolution to break down thermosetting polymers containing ester groups, enabling the recovery of electronic components from PCBs. Mir and Dhawan^77^ reviewed the current commercial-scale recycling technologies for PCBs. They also suggested a futuristic recycling perspective and value-added products from waste PCBs. Zhu et al.^78^ summarized the studies showing the potential of waste PCB using pyrolysis. Cozza et al.^79^ proposed a circular manufacturing ecosystem for the automotive PCB. A major challenge lies in the non-metallic fraction of waste PCBs, which constitutes roughly 60 wt % of the total and has relatively low economic value. From an environmental management standpoint, adopting a zero-waste approach to recycling this fraction could help recover value. Wang et al.^80^ reviewed the studies focusing on the utilization of non-metallic PCB waste in polymer composites and geopolymers. In conclusion, the pursuit of sustainable and carbon-neutral solutions for PCBs has seen significant progress through various R&D initiatives. Feasibility and constraints: recycling innovations only reduce scope 3 demand if they operate at scale and displace virgin metals/laminates. Otherwise, they remain end-of-life treatment rather than a circular supply. The non-metallic fraction is the structural bottleneck for circularity, which is why design-for-recycling and material choices should be treated as enabling conditions for high-yield recovery rather than optional add-ons.

The most “deployable” near-term decarbonization measures are those that align with known hotspots (energy/electricity use, wet-process chemistry, and copper/material intensity) and that also satisfy qualification, cost, and end-of-life system constraints. This sets up the pathway synthesis in later sections by distinguishing what is technically promising from what is scalable under real supply-chain conditions.

### Unified quantitative comparison and feasibility assessment of decarbonization measures

Recent synthesis studies on industrial decarbonization and circular economy transitions consistently show that electricity decarbonization delivers the largest near-term absolute emission reductions, while material circularity, recycling, and process redesign primarily enable medium-to long-term scope 3 mitigation under favorable system and governance conditions. For example, system-level analyses of manufacturing decarbonization emphasize the dominant role of energy supply and efficiency in near-term mitigation, with circular economy strategies constrained by material complexity, infrastructure availability, and rebound effects.^81^^,^^82^^,^^83^ The PCB-specific evidence synthesized here aligns with these broader findings but reveals additional constraints unique to PCB manufacturing, including multilayer board complexity, qualification and reliability requirements, and limited end-of-life separability of metal-polymer composites. These factors motivate the unified, pathway-level comparison presented in the further text.

Despite the growing number of sustainability initiatives across the PCB supply chain, reported decarbonization outcomes are frequently presented as isolated, case-specific claims, limiting cross-study comparability. To address this gap, this section synthesizes representative PCB decarbonization measures using a unified evaluation framework considering (1) life cycle stage targeted, 2) dominant emissions scope (scope 1–3), (3) reported carbon-reduction magnitude, (4) technological maturity, and (5) feasibility constraints and potential trade-offs. These representative decarbonization measures, reported reduction ranges, technological maturity levels, and feasibility constraints are consolidated into a boundary-qualified comparative framework (Table 3).

**Table 3:** Technology-level comparison of representative PCB decarbonization measures (evidence-tiered and boundary-qualified)

Measure (representative)	Life cycle stage	Main scope affected	Reported quantitative effect (as stated in source)	Evidence tier	Key boundary/feasibility caveats
Renewable electricity/decarbonized purchased power (example PCF^40^)	manufacturing	scope 2	in a 10-layer PCB ISO 14067 PCF, manufacturing = **58.17%** of total and outsourced electricity = **92.81%** of manufacturing-stage footprint (indicating electricity as dominant lever for that product/system).	third-party PCF/audited disclosure	• reduction magnitude depends on the local grid emission factor and the procurement mechanism • not directly transferable across regions.
Substrate substitution (Soluboard® example^52^^,^^54^^,^^84^^,^^85^)	materials/EoL enabling	scope 3	Soluboard® reported **∼60%** carbon-emission reduction vs. FR-4 (case-specific; per m^2^).	company/partner disclosure	dependent on-board spec, boundary, and qualification + end-of-life pathway (delamination/collection).
Inkjet/semi-additive fabrication (Elephantech example^86^)	fabrication	scope 2 + process burdens	manufacturer claims **∼75% CO**_**2**_, **∼70% copper**, **∼95% water** reduction vs. conventional (product-specific).	company-reported	treat as case-specific benchmark unless verified; limited product classes; multilayer/high-density interconnect (HDI) scalability constraints.
Facility LCA hotspot (Ozkan plant study^39^)	fabrication	scope 2 + toxicity categories	• PCB production contributes **∼89%** freshwater aquatic ecotoxicity potential and **∼73%** ozone depletion potential. • etching is the primary hotspot across most categories. • etching ∼**33%** of manufacturing-stage global warming potential (facility case).	peer-reviewed LCA	• facility- and geography-specific; category contributions are not universal. • illustrates hotspot directionality.
Design parameters and hotspot sensitivity (Ochoa et al.^42^)	design → manufacturing	scope 2 + materials	cradle-to-gate impacts dominated by manufacturing energy + laminate materials (together **∼80%**, design-dependent).	peer-reviewed LCA/design study	strong dependence on board design parameters, yield assumptions, and electricity EF.
Product-level context (Fairphone 5^43^)	product assembly context	scope 3 upstream	• primary PCBA **17.8 kg CO**_**2**_**eq.** • ICs dominate within PCBA (16.2 kg), PCB itself 1.47 kg (module-level).	product LCA	• device-specific. • used here only to link PCB→ product footprint and scope prioritization.
Low-temperature soldering (LTS) in Surface Mount Technology (SMT) lines (Winbond example^87^)	assembly (PCBA)	scope 2 (electricity/thermal energy)	Winbond reports LTS lowers SMT temperature to ∼190°C and reduces CO_2_ emissions by **57 metric tons per year per SMT production line**.	company ESG report/disclosure	• line-level operational reduction. • not a full PCF/LCA. • depends on line utilization, electricity mix, and baseline reflow profile.
Recycled tin solder + recycled gold plating in PCBs (Apple-designed PCBs^60^^,^^88^)	materials (upstream)	scope 3 (upstream materials)	• Apple states that by 2025 it will use **100% certified recycled tin soldering** on all Apple-designed rigid and flexible PCB.^60^ • Apple also commits to **100% recycled gold plating** in Apple-designed rigid and flexible PCB by 2025.^88^	Corporate disclosure (Apple)	• provides recycled-content commitments/coverage, not a quantified CO_2_ delta. • carbon impact depends on the metal supply-chain EF and the allocation method.

This table presents boundary-defined examples rather than pooled averages. Values are not directly comparable unless the functional unit, boundary, and electricity assumptions are harmonized. The evidence tier indicates whether values are peer-reviewed, audited PCF, or company-reported. Here, PCF: product carbon footprint, LCA is life cycle assessment, LTS: Low-temperature soldering, SMT: Surface Mount Technology, PCB: printed circuit board, PCBA: printed circuit board assembly, ISO: International Organization for Standardization, FR-4: Flame retardant, HDI: high-density interconnect.

Electricity-related measures, including renewable electricity procurement, energy-efficiency improvements, and process electrification, consistently exhibit the highest near-term mitigation potential. ISO 14067-based product carbon footprint disclosures and facility-level LCAs indicate that electricity consumption can account for 80%–95% of manufacturing-stage emissions for multilayer PCBs under fossil-dominated grid mixes, implying that grid decarbonization alone can reduce cradle-to-gate emissions by 40%–70%, depending on regional electricity intensity (Table 3).^39^^,^^40^^,^^43^ Material substitution strategies, such as bio-based laminates (e.g., Soluboard), recycled metals, and reduced copper foil thickness, primarily target scope 3 upstream emissions embedded in copper and laminate production. Reported reductions typically range from 30% to 60% per square meter of PCB, but outcomes are highly boundary-dependent and sensitive to qualification requirements (Table 3) and assumed end-of-life pathways.^54^^,^^89^ For example, while Soluboard demonstrates substantial embodied carbon reduction under controlled conditions, its net decarbonization potential at scale depends on moisture resistance, solder-reflow compatibility, and the availability of delamination-enabled recovery infrastructure.

Process redesign approaches, including additive or semi-additive PCB fabrication and low-temperature soldering, offer intermediate reductions (10–40%) by reducing copper losses, chemical consumption, and thermal energy demand.^58^^,^^71^ However, scalability remains constrained by throughput, yield stability, multilayer capability, and compatibility with high-density interconnect designs, and several reported reductions rely on manufacturer data without third-party life cycle verification. End-of-life-oriented strategies, such as design-for-disassembly and advanced recycling, primarily influence long-term scope 3 emissions by reducing virgin material demand. Although laboratory studies report high metal recovery efficiencies, system-level carbon benefits remain contingent on collection rates, logistics, and recycling energy intensity.^77^^,^^79^ In regions dominated by informal recycling, design-for-recycling improvements may yield limited realized climate benefits. Therefore, this comparative assessment demonstrates that no single decarbonization measure is sufficient to achieve net-zero PCB manufacturing. High-impact near-term reductions are most reliably achieved through electricity decarbonization and energy efficiency, while material innovation, process redesign, and circular strategies play enabling roles for medium-to long-term scope 3 mitigation.

## Strategies for decarbonizing the PCB supply chain

**Figure 4:**
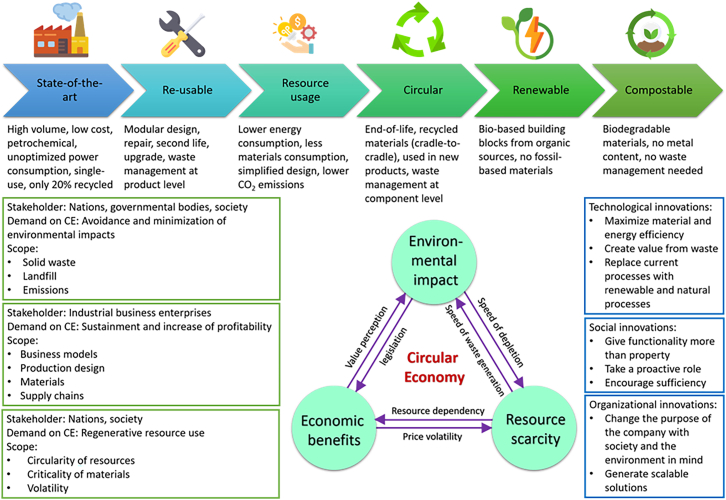
Pathways and strategies for decarbonizing PCB supply chain, incorporating circular economy principles between governments, society, and the business sector in the PCB supply chain^94^ Credit: www.flaticon.com for the clipart.

Since the PCB is an essential component of a wide range of electronic devices, the PCB supply chain has an important role to play in achieving global decarbonization and the transition to a low-carbon economy. This supply chain is complex and global, involving numerous actors and stages, from raw materials extraction through to the finished product. There are numerous challenges to decarbonizing its supply chain. Firstly, the industry is highly energy-intensive, and this is mainly due to the process of drilling and etching the copper on the boards, as well as other processes such as soldering, surface finishing, and packaging. Another challenge is the lack of transparency in the supply chain. Many of the suppliers, manufacturers, and distributors of PCBs are based in countries with lower environmental standards, and it is difficult to accurately track the carbon emissions of each stage of the supply chain. This lack of transparency makes it difficult to accurately assess the carbon footprint of the industry, as well as implement effective decarbonization strategies. Finally, the changing nature of the industry also poses a challenge to decarbonizing the PCB supply chain. Therefore, this study proposes five key strategies for decarbonizing the PCB supply chain: (1) renewable energy sources and adoption of energy-efficient production processes, (2) sustainable materials and design optimization, (3) promotion of circular economy practices and carbon offset programs, (4) LCA, and (5) development of industry standards and regulations. To address cross-study comparability and avoid case-listing, Table 4 summarizes a unified evaluation framework that links each pathway to life cycle hotspots, relevant emission scopes, evidence-based reduction ranges (where available), maturity/readiness, and key feasibility constraints. Figure 4explains the model of Lieder and Rashid,^94^ which illustrates the commitment and interaction that must exist between governments, society, and the business sector, and how it can play a vital role in the adoption of circular economy principles to achieve carbon neutrality in the PCB supply chain. Operationally, these interactions translate into government-driven standards and renewable-energy policy shaping scope 2 reductions. Societal procurement and consumer pressure increasing scope 3 transparency and industry-led design-for-circularity enabling higher end-of-life recovery and closing material loops. A technology-level comparison of representative PCB decarbonization measures, including reported reduction ranges, maturity levels, and feasibility constraints, is provided in Table 3.

**Table 4:** Pathway-level synthesis and feasibility assessment for PCB decarbonization

Pathway	Primary life cycle hotspot(s) addressed	Main emission scope(s) affected	Quantitative evidence/indicative reduction ranges (boundary-dependent)	Maturity/readiness (qualitative)	Key feasibility constraints
(1) Renewable electricity + energy efficiency	• manufacturing electricity • facility utilities	scope 2 (and parts of scope 1 via electrification)	product carbon footprint disclosures show manufacturing dominated by electricity in some multilayer boards (e.g., manufacturing share ∼58% and outsourced electricity contributed ∼93% of the manufacturing-stage footprint in an ISO 14067-based PCF), implying large reductions are achievable when grid/electricity supply is decarbonized.^40^	commercial (technology), deployment varies by region	• access to low-carbon electricity/PPAs and credible contractual instruments. • site-level grid constraints. • Capex for efficiency retrofits. • dual-reporting/accounting complexity for Scope 2.
(2) Sustainable materials + design optimization (e.g., laminate substitution, recycled content, reduced plating)	• laminate/resin systems • metal intensity (Cu/Au/Sn) • design-driven yield/scrap	scope 3 (upstream materials), plus indirect Scope 2 via yield improvements	• example: replacing FR-4 with Soluboard® reported ∼60% carbon-emission reduction and material savings per m^2^ (case-specific • depends on boundary and board spec).^52^^,^^54^^,^^84^^,^^85^	pilot → early commercial (application-dependent)	• qualification/reliability (thermal cycling, moisture, solder reflow). • compatibility with multilayer/HDI. • cost premium. • verified chain-of-custody for recycled metals.
(3) Process redesign & additive/printed approaches (reducing subtractive etching)	• etching/copper loss • wet processing • wastewater and sludge burdens	scope 2 (energy), scope 1 (process energy/chemicals), plus waste-related burdens	example: Elephantech reports ∼75% CO_2_ reduction, ∼70% lower copper use, and ∼95% lower water use for its inkjet/plating approach (company-reported; treat as case-specific unless independently verified).^86^	pilot → commercial in limited product classes	• throughput and multilayer capability. • yield/defect control. • HDI compatibility. • supply qualification by OEMs. • comparability of claims without standardized PCF boundaries.
(4) Circular economy strategies (design-for-disassembly, collection, recycling/recovery, targeted offsetting where unavoidable)	• virgin-material displacement • end-of-life separation • non-metallic fraction bottleneck	scope 3 (downstream and upstream displacement effects)	reduction magnitude is highly variable and system-dependent; benefits depend on whether recovery actually displaces virgin metals/laminates at scale (i.e., not just “treatment”).	• mixed • many routes are regional and infrastructure-limited	• collection and take-back systems. • economics and purity requirements. non-metallic fraction management. • regulatory enforcement. • risk of burden shifting (toxicity/energy trade-offs).
(5) Systematic LCA + PCF	• cross-stage hotspot identification • prevents “apples-to-oranges” comparisons	enabling across Scopes 1–3	ISO 14067 provides the product carbon footprint framework; multiple PCB PCF certificates/reports show declared-unit and boundary sensitivity, supporting your “range + boundary-qualified” approach^15^^,^^40^^,^^90^	• mature method • data availability uneven	• supplier data opacity. • inconsistent functional units and boundaries. • limited representativeness. • verification cost.
(6) Harmonized standards + net-zero governance (supplier engagement, reporting, claims integrity)	supply-chain alignment; prevents “paper decarbonization”	enabling across Scopes 1–3	• the Science Based Targets initiative (SBTi) guidance requires Scope 3 targets and provides supplier-engagement mechanisms • ISO 14068-1 sets requirements for carbon neutrality management plans and treatment of residual emissions/offsetting, improving credibility of net-zero claims.^91^^,^^92^^,^^93^	emerging/rapidly evolving	• regional mismatch (regulation vs. manufacturing geography). • assurance/verification burden • inconsistent claim quality. • supplier engagement capacity limits.

Reduction values are not directly comparable across initiatives unless functional unit, system boundary (cradle-to-gate vs. cradle-to-grave), board specification (layer count/material stack), yield, and electricity factors are aligned. Values above are therefore reported as boundary-dependent indicators rather than universal benchmarks.

Here, PCF: product carbon footprint, LCA is life cycle assessment, Cu: Copper, Au: Gold, Sn: Tin, SBTi: Science Based Targets initiative, ISO: International Organization for Standardization, PPA: Power Purchase Agreement, HDI: high-density interconnect, OEM: Original Equipment Manufacturer.

Consistent with recent cross-sector decarbonization syntheses, the strategies proposed here distinguish near-term, electricity-driven reductions from longer-term circularity-enabled mitigation, while explicitly accounting for feasibility, governance, and system-boundary constraints that are often underrepresented in generic circular economy frameworks.^81^^,^^82^^,^^83^

### Sustainable materials and design optimization

The optimization of PCB designs marks a foundational step in the supply chain, with design for manufacturing (DFM) and design for assembly (DFA) forming the critical DFMA link between design and manufacturing processes. This approach, which minimizes costs and production time while enhancing product quality and reliability, requires adherence to industry certifications like AS9100D/ISO9001:2015 and RoHS. Beyond design, a key strategy for improving product sustainability involves prioritizing repairability to extend end-of-life. Incorporating features such as modular designs with repairable or replaceable components, or enabling upgrades, contributes significantly to the environmental impact by negating the need for premature replacements. This integrated approach aligns with the broader goal of reducing the environmental footprint throughout the product’s life cycle, showcasing a commitment to sustainable practices in the production of high-quality PCBs. Most importantly, design and material optimization primarily reduce scope 3 upstream emissions, while secondary reductions in scope 1 emissions may occur when design simplification enables fewer or lower-temperature manufacturing steps. Simplified designs with reduced coatings can significantly lower material usage, shorten production steps, and reduce energy demand. Some design optimization considerations are increasing circuit density through saving space on the substrate, decreasing the weight of the product, improving electrical properties (additional termination and shortening electrical connections), increasing manufacturing automation, and eliminating incorrectly attached devices and solder joints.

Adopting new technologies can open the door to additional innovations, such as shifting to alternative materials and additive manufacturing techniques. In this context, flexible PCBs are becoming a key component of the evolving electronics sector, offering more diverse applications than traditional rigid designs. Their fabrication often requires rethinking conventional methods, for instance, substituting plastic or paper for the standard FR substrates. A comparison of organic-based and paper-based PCBs is presented in Table S3. When working with plastic substrates like poly(ethylene terephthalate), which have lower thermal resistance, low-temperature processing becomes particularly important. In such cases, electrically conductive adhesives present a better alternative to lead-based solder, removing the need for flux cleaning and simplifying the overall manufacturing process.^72^

Achieving a circular use of materials necessitates significant changes, especially in enabling alternative choices at end-of-life. Dismantling products into their chemical building blocks becomes crucial for circularity. Printed electronics offer advantages here, as many starting materials are meltable, facilitating dismantling. While the cradle-to-cradle recycling approach is favored, challenges arise with integrated electronic products designed for durability. Disassembly becomes difficult, leaving shredding and incineration as the only end-of-life options. Plastics in these products serve as energy carriers for metallurgical processes, but achieving high purity levels for recycling poses challenges. Design-for-recycling principles are vital for PCB production in a circular economy, ensuring the disassembly of products for optimal component and material reuse, leading to higher recycling rates. Some of the large-scale circular materials and bio-based PCBs are discussed in process overview.

Artificial intelligence and the IoT play a significant role in advancing sustainable manufacturing. Smart digital manufacturing techniques, such as process automation and the use of sensors to detect leaks or inefficient material usage, enable companies to reduce waste and lower unnecessary expenses. Data-driven analysis can further streamline operations by identifying redundant steps and highlighting areas where material and energy consumption can be minimized. This shift toward digitization is gaining momentum, with many major brands adopting similar strategies. For instance, Sharma and Kumar^95^ implemented a computer vision system to enhance the recycling of PCB components. In late 2021, Apple announced its participation in sustainable semiconductor technologies and systems, an initiative led by the Belgian research institute Imec, aimed at reducing the environmental footprint of semiconductor production. The program leverages digital tools to identify potential improvements in energy use, GHG emissions, and water consumption. Other major companies, such as Microsoft, Amazon, and ASML, are also reported to utilize Imec’s analytical methods.

In addition, additive manufacturing approaches offer the potential to reduce both costs and emissions by eliminating the excess materials and etching processes inherent in traditional subtractive techniques. For instance, adopting additive PCB manufacturing methods can decrease water usage by as much as ∼95%,^96^ which may translate into indirect carbon reductions depending on the electricity intensity of water treatment and wastewater handling. Additive manufacturing is not only energy-efficient due to its operation at low temperatures, but it is also characterized by rapid production capabilities. This dual advantage makes additive manufacturing, often referred to as 3D printing, an attractive and efficient technology.

### Renewable energy sources and adoption of energy-efficient production processes

The PCBs and renewable energy industries are inextricably interdependent. The fossil fuel-based energy sources of PCB manufacturing could be replaced by renewable energy sources such as hydroelectric, solar, wind, and geothermal power, based on the geographical location and available resources. This green and clean energy could be deployed to PCB manufacturing industries to power the equipment used in various stages, from designing and printing to etching and assembly. More and more companies are seeking to power their new fabrication plants with renewable energy. Samsung, for example, reports 100% renewable electricity procurement for its facilities in the US and China through power purchase agreements and renewable energy certificates.^96^ Similarly, companies like Apple, IBM, Intel, and Nokia have begun integrating renewable energy into their PCB manufacturing operations. However, the accessibility of renewable energy differs significantly by region. In areas such as the US and Europe, where renewable energy resources are more readily available, this has contributed to renewed interest in local PCB production in some regions.^96^ Additionally, the participation of PCB manufacturing companies in renewable energy plans and carbon trading markets could help achieve scope 2 (indirect emissions from energy usage) carbon reduction in net-zero emissions targets and meet regulatory requirements.

Under the EU’s “Fit for 55” legislative package, binding targets have been introduced to substantially increase the share of renewable energy and reduce GHG emissions by at least 55% by 2030.^97^ One of the 55 regulations in the first European Climate Law is to increase the production of renewable energy sources by 8% by 2030.^97^ On the flip side, PCBs play a pivotal role in renewable energy systems by facilitating the functionality of clean technology. PCBs offer dependable electrical connections, contribute to the compact and scalable nature of renewable energy solutions, enable the miniaturization of electronic components, streamline integration into complex systems, and provide enhanced durability and reliability in challenging environmental conditions. Consequently, it becomes imperative for the PCB industry to undergo decarbonization, as its sustainability is intricately linked to the overall sustainability of the renewable energy sector.

As energy prices rise globally, energy-efficient measures, including advanced printing technologies, optimizing production lines, and waste reduction, become crucial for decarbonized PCBs. Significant energy savings and improved energy self-sufficiency are expected to make the manufacturing of various electronic components increasingly cost-competitive in the Asia-Pacific region. Indicative facility-level energy-saving potentials reported for electronics manufacturing include waste heat and energy recovery (35%); improvements to boilers, fired systems, process heating, and cooling (17%); energy source flexibility and use of CHP (16%); improved sensors, controls, automation, and robotics for energy systems (4%); and energy system integration and best practice opportunities (28%).^98^ These values represent indicative potentials reported for industrial manufacturing systems and should be interpreted as order-of-magnitude guidance rather than PCB-specific guarantees.

In this context, collaboration between government agencies, renewable energy providers, and industry organizations can help leverage resources, incentives, and technical expertise for renewable energy adoption. For instance, the Institute for Factory Automation and Production Systems (FAPS), through the Green Factory Bavaria project, is working to reduce energy consumption at every stage of the PCB assembly process. Similarly, the SurfEnergy project offers a structured framework for improving energy efficiency, including guidance on establishing an energy management system, meeting energy auditing requirements, compiling measure lists, using tools to assess energy performance, and making informed investment decisions.^72^ These measures primarily target electricity-dominated manufacturing hotspots (environmental impact) and scope 2 emissions, consistent with the pathway-level synthesis in Table 4.

### Promotion of circular economy practices and carbon offset programs

China stands as the global leader in PCB manufacturing, boasting a remarkable output value of USD ∼77.7 billion in 2021.^99^ A substantial share of PCBs manufactured in China is exported, contributing to long-distance transportation emissions. The transportation of PCBs over long distances, especially by air, can contribute significantly to carbon emissions. Moreover, proper packaging is essential to prevent damage during transportation, as inadequately packaged PCBs pose the risk of generating e-waste and releasing hazardous materials into the environment. Opting for local PCB sourcing minimizes transportation needs, reducing emissions by covering shorter distances. Local manufacturing not only ensures transparency in the supply chain but also provides insights into sustainability practices, guaranteeing adherence to environmental regulations. This approach underscores the importance of localizing PCB production for both environmental and supply chain management benefits. However, the net carbon benefit of localization depends on the electricity mix, production efficiency, and logistics mode, and is not guaranteed in regions with carbon-intensive grids.

Over the past three years, the electronics industry has faced considerable turbulence, driven by pandemics, trade conflicts, and energy crises, all of which have underscored its vulnerability. The global semiconductor shortage serves as a clear example of how disruptive supply chain breakdowns can be. In response, there is a growing push to redistribute manufacturing capacity, with major investments supporting the revival of localized electronics production in Western regions, as outlined in the US CHIPS & Science Act and the European Chips Act.^100^^,^^101^ The trend toward “reshoring” creates a valuable opportunity to integrate sustainability principles from the ground up when designing and equipping new manufacturing facilities. By contrast, existing production lines face greater hurdles, as much of their equipment is already depreciated, making the relative cost of transitioning to more sustainable production methods significantly higher.

Beyond sustainable logistics and localized production, design for recycling (DfR) represents a critical circular-economy lever. Implementing DfR enhances material recovery, one of the circular economy practices. Comparative studies favor manual PCB extraction over automated shredding, citing significantly higher recovery rates (95%−99% vs. 12%−60%).^102^ Extensive dismantling followed by high-quality recycling, as supported by research, leads to the most substantial reduction in global warming potential of the product. Norgren et al.^103^ exploration of DfR across use cases emphasizes its role in increasing the quantity and value of materials recovered from end-of-life products. We summarized a list of degradable materials used in an electronic device and key design guidelines for recycling and reuse of PCBs in Tables 5 and 6.

**Table 5:** List of degradable materials used in an electronic device

For substrates	For conductors	For dielectric	Semiconductor
Natural polymers	(Electro)chemically doped conjugated polymers	Natural	Organic/Natural
• Paper • Nano-cellulose • Silk • Shellac • Gelatin • Chitosan • Silk fibroin—Polyvinyl alcohol composite	• paper • nano-cellulose • silk • shellac • gelatin • chitosan • silk fibroin—polyvinyl alcohol composite	• paper • nano-cellulose • silk • shellac • gelatin • chitosan • silk fibroin—polyvinyl alcohol composite	• indigo • melanin • tyrian purple • acridones • anthraquinones • terpenoids • phenazines • perylene diimide • beta carotene

**Synthetic polymers**	**Metal**	**Synthetic polymers**	**Synthetic polymers**

• Polyvinyl alcohol • Polylactic acid, Poly-L-lactic acid • Polycaprolactone-flexible • Polyurethane • Polybutylene succinate • Polyethylene glycol • Poly-lactic-co-glycolic acid • Polydimethylsiloxane • Poly(hydroxybutyrate-co-valerate)	• molybdenum • ferrum • tungsten • zinc • magnesium	• polyvinyl alcohol • polylactic acid • polyurethane • polydimethylsiloxane • poly (lactic-co-glycolic acid)- polycaprolactone composite	• polythiophenes • poly (3-hexylthiophene)

Metal	Inorganic	Inorganic
• Molybdenum • Ferrum • Tungsten • Zinc • Magnesium	• magnesium oxide • silicon dioxide • silicon nitride • spin-on-glass	• silicon nano membrane • polycrystalline silicon • amorphous silicon • germanium • silicon-germanium alloy • indium-gallium-zinc oxide

Adapted from Chakraborty et al.^104^

**Table 6:** Key design guidelines for recycling and reuse of PCBs

Key design guidelines for recycling and reuse of PCBs	
**Avoid**	

• Design simple to disassemble and recycle • Use recycled materials • Reduce size and minimize the consumption of materials • Minimize the use of magnets or magnetic components • Fix printed circuit boards with screws, press fit, or connectors • Use removable batteries, avoid glue or welding	• halogenated polymers like polyvinyl alcohol, polytetrafluoroethylene, etc. • toxic additives • galvanization or metallization on plastic • coating by painting or lacquering • the use of non-recyclable thermosets, elastomers • mixing of ferrous and non-ferrous metals • use of brominated flame retardants in product design

Separation method	Principle of separation	Separated elements
**Methods for the separation of elements**		

Uncontrolled incineration	combustion	non-metallic part is removed
Mechanical separation	depends on the material properties	precious metals
Gravity separation	specific gravity	plastics, resins, glass, from heavy metals
Pyrometallurgy	thermochemical treatment	mineral and metallic phases from plastic
Hydrometallurgy	strong acid treatment	selectively dissolve and precipitate metals
Magnetic separation	magnetic susceptibility	ferrous metals from nonferrous metals
Electrostatic separation	electric conductivity	ferrous metals from nonferrous metals

**Leaching agents and recovered metals from waste PCBs**		

Solder metal leaching	Na1OH, HCl, H_2_SO_4_, NaCl, HBF_4_, SnCl_4_-HCl, Fe_2_(SO_4_)_3_-H_2_SO_4_, and HNO_3_	Pb, Sn, Zn, Fe
Base metal leaching	H_2_SO_4_, H_2_O_2_, HCl, HNO_3_, EDTA, citrate, H_2_SO_4_-CuSO_4_-NaCl, Fe_2_(SO_4_)_3_-H_2_SO_4_, HCl-FeCl_3_	Cu, Au, Fe, Zn, Ni, Al, Ag, Sn
Precious metal leaching	(NH_4_)_2_S_2_O_3_, CuSO_4_, NH_4_OH, NaCN, H_2_SO_4_, thiourea, H_2_O_2_, iodine-iodide lixiviant, aqua regia	Au, Ag, Pd

Here, NaOH: Sodium hydroxide, HCl: Hydrochloric acid, H_2_SO_4_: Sulfuric acid, NaCl: Sodium chloride, HBF_4_: Tetrafluoroboric acid, SnCl_4_: Tin(IV) chloride, Fe_2_(SO_4_)_3_: Ferric sulfate, HNO_3_: Nitric acid, H_2_O_2_: Hydrogen peroxide, EDTA: Ethylenediaminetetraacetic acid, CuSO_4_: copper(II) sulfate, FeCl_3_: ferric chloride, (NH_4_)_2_S_2_O_3_: Ammonium thiosulfate, NH_4_OH: Ammonium hydroxide, NaCN: Sodium cyanide, Pb: Lead, Sn: Tin, Zn: Zinc, Fe: Iron, Cu: Copper, Au: Gold, Ni: Nickel, Al: Aluminum, Ag: Silver, Pd: Palladium.

Adapted from Chakraborty et al.^104^

DfR facilitates the metal recovery from waste and discarded PCBs. Metal production is responsible for 8% of the total global energy consumption. Relative to primary material production, reported estimates indicate that recycling 10 kg of Cu, Fe & steel, Pb, Zn, paper, and plastics can save approximately 85%, 74%, 65%, 60%, 64%, and <80% of energy, respectively. This could be saved at the material production stage, assuming recycled material directly displaces primary production.^104^ By recycling 10 kg of Al, it is possible to save 90% of energy and prevent the creation of 13 kg of bauxite residue, 20 kg of CO_2_ gas, and 0.11 kg of SO_2_ gas.^104^ Indeed, recovering metals from PCBs would effectively mitigate the emissions associated with metal mining and processing. R&D is also going on in the direction of making PCB recycling cost-effective, easier, and less carbon- and energy-intensive. For example, the energy demand of chemical recycling routes for waste PCBs can be reduced by optimizing degradation temperatures and reaction media for cured thermosetting resins.^105^

In the cases where emissions are often necessary, reducing environmental impact can be challenging. Carbon offsetting does not reduce the calculated product carbon footprint under ISO 14067,^15^^,^^106^ but may be applied only to residual emissions after reduction measures when pursuing carbon-neutrality claims under ISO 14068.^91^ However, alternative pathways can be adopted, such as purchasing high-quality carbon offsets or supporting external projects with substantial emission reductions. Carbon offsets involve compensating for emissions by reducing or removing an equivalent amount of carbon dioxide. This can include sponsoring initiatives like solar energy farms or reforestation projects in vulnerable areas. Notably, since the start of 2022, M-TEK has successfully attained a net-zero carbon claim by planting a tree in Uganda for every PCB manufactured by M-TEK assembly.^107^ Such offset-based approaches do not substitute for direct emission reductions and are most appropriately applied to residual emissions after feasible scope 1–3 mitigation measures have been implemented.

**Figure 5:**
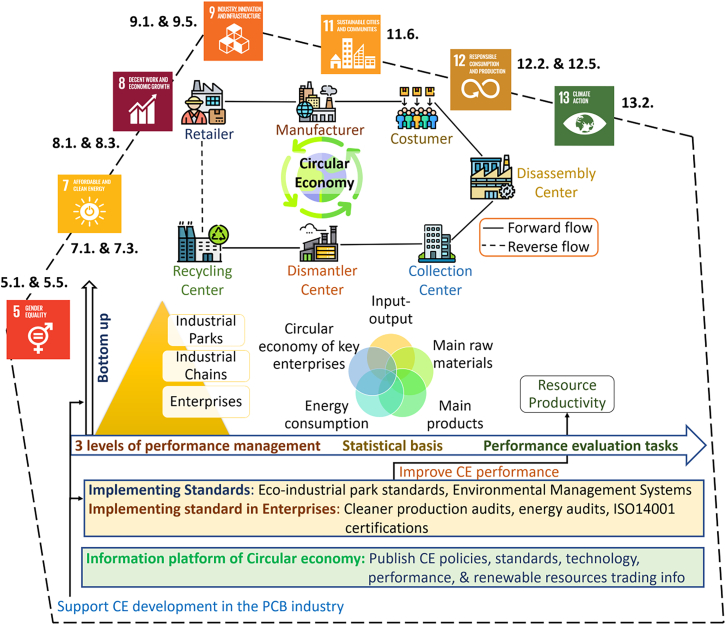
Circular economy management model for the PCB industry to achieve the SDGs A part of the figure is adapted from Wen and Meng^109^ and recreated with additional data to make this figure. Credit: www.flaticon.com for the clipart.

The PCB manufacturing industry could also use eco-efficiency indicators, which are the revenue created by each unit of pollutant emissions and resource consumption. For example, energy usage (30 USD/1000 kWh), water usage (30 USD/metric tons), GHG emissions (30 USD/kgCO_2_eq), waste (30 USD/kg), and wastewater (30 USD/metric tons).^108^ Subsequently, Figure 5 illustrates a circular economy management model in which material efficiency, recycling, and localized production reduce life cycle emissions within the product carbon footprint, while offsetting is treated as a complementary mechanism applied only to residual emissions. Together, localization, DfR, high-quality recycling, and limited use of offsets primarily influence long-term scope 3 emissions by reducing virgin material demand and logistics-related burdens.

### Implementation of life cycle assessment

This study follows the greenhouse gas accounting and reporting requirements of ISO 14064-1^14^ and ISO 14067:2018.^110^ In accordance with these standards, emissions are classified using ISO categories 1–6 rather than the scope 1–3 structure defined in the GHG protocol. Category 1 covers direct emissions from owned or controlled sources, category 2 covers indirect emissions from purchased energy, and categories 3–6 encompass other indirect value-chain emissions. Where scope-based terminology (scope 1–3) is used elsewhere in this article, it is applied solely as an interpretive mapping for readability and alignment with common industry discourse, and not as the underlying accounting framework. All quantitative product carbon footprint results reported in this study are calculated and interpreted in compliance with the ISO 14067 system boundary, allocation, and reporting requirements.

Quantification of environmental impacts in the PCB supply chain is most rigorously conducted using LCA. LCA is particularly important for evaluating whether proposed material substitutions, process redesigns, or circular strategies deliver genuine environmental benefits rather than burden shifting. While attributional LCA is most commonly applied in PCB studies, consequential LCA and ecological carbon footprint approaches are also discussed in the literature; however, the quantitative framework adopted here follows an attributional product carbon footprint approach consistent with ISO 14067.^106^

The PCB supply chain is assessed across its full life cycle, including raw material extraction and processing, board fabrication and assembly, distribution, use, and end-of-life management. The total product carbon footprint is therefore expressed as (Equation 1):

(Equation 1) $$CF_{Total}=CF_{RM}+CF_{M}+CF_{D}+CF_{U}+CF_{EOL}\text{,}$$

where *CF*_RM_ is the carbon footprint of the raw material stage (kg CO_2_ eq); *CF*_M_ is the carbon footprint of the manufacturing stage (kg CO_2_eq); *CF*_D_ is the carbon footprint of the distribution stage (kg CO_2_eq); *CF*_U_ is the carbon footprint of the use stage (kg CO_2_eq); *CF*_EOL_ is the carbon footprint of the end-of-life stage (kg CO_2_eq).^106^

In practice, product-specific metering data are rarely available in PCB fabrication facilities. Consequently, many studies allocate manufacturing emissions using facility-average approaches. The total manufacturing carbon footprint of a single PCB within a product, $MCF_{PCB}$ (kgCO_2_eq), can be estimated using an area-based allocation model^111^:

(Equation 2) $$MCF_{PCB}=\left[\left(\frac{GHG_{F}}{A_{layer\_total}}\right)\right]\times \left(A_{PCB\_Board}\times L_{PCB}\right)\text{,}$$

where $GHG_{F}$ is the total manufacturing facility GHG emissions in a year (kgCO_2_eq), $A_{layer\_total}$ is the total area of layers of board produced in the facility for that year (m^2^), $A_{PCB\_Board}$ is the specific PCB area for the product being studied (m^2^), and $L_{PCB}$ is the layers of PCB for the product being studied.

Equations 2 and 3 represent a facility-average, area-based allocation approach commonly used when product-specific energy metering is unavailable. This method assumes comparable process routes and energy intensity across PCB layers and products. Therefore, it provides an approximate estimate suitable for benchmarking and hotspot identification rather than precise product-specific accounting.

To distinguish between direct and indirect manufacturing emissions, the manufacturing carbon footprint can be further separated into scope 1 and scope 2 contributions (Equation 3)^111^:

(Equation 3) $$MCF_{PCB}=\left[\frac{GHG_{scope1}}{\left(A_{board\_total}\right)}\right]\times \left(A_{PCB\_Board}\right)+\left[\frac{GHG_{scope2}}{\left(A_{layer\_total}\right)}\right]\times \left(A_{PCB\_Board}\times L_{PCB}\right)\text{,}$$

where, $GHG_{scope1}$ is the total scope 1 manufacturing facility GHG emissions in a year (kgCO_2_eq), $GHG_{scope2}$ is the total scope 2 manufacturing facility GHG emissions in a year (kgCO_2_eq), $A_{layer\_total}$ is the total area of layers (inner and outer) of board produced in the facility in that year (m^2^), and $A_{board\_total}$ is the total area of board produced in the facility in that year (m^2^). This distinction reflects the fact that scope 2 emissions are typically driven by layer-dependent processes such as lamination, drilling, and plating, whereas scope 1 emissions are more closely associated with facility-wide thermal processes that scale with total board output.^106^^,^^111^ Upstream scope 3 emissions from materials, chemicals, and outsourced processes are not included in Equations 2 and 3 and must be accounted for separately using supplier-specific data or secondary life cycle inventory datasets. This limitation contributes substantially to the variability observed across reported PCB carbon footprints.

Consistent with this methodological heterogeneity, published LCAs and ISO 14067-based product carbon footprint disclosures report wide ranges in global warming potential depending on functional unit, system boundary, board complexity, yield assumptions, and electricity mix (Table 2). As a result, PCB carbon footprint values are most appropriately interpreted as boundary-qualified ranges rather than single representative numbers.

Guidelines for reducing environmental impacts in printed electronics further illustrate the leverage of design and material choices. Prenzel et al.^112^ provided strategies for reducing embodied emissions through conductor and substrate selection or example, substituting copper for silver in printed circuitry can reduce global warming potential by more than 95%, although this benefit is constrained to the circuitry itself if other processing steps remain unchanged.^113^ Similar reductions may be achieved through alternative substrate materials (e.g., paper-based carriers), albeit with trade-offs in moisture sensitivity and reliability. At higher integration levels, ultra-thin organic photovoltaic devices demonstrate reported carbon footprint reductions of 10%–85%, while in-mold structural electronics achieve reductions of 34%–62% relative to conventional PCBs, depending on application-specific assumptions.^114^ Taken together, these findings reinforce that LCA and product carbon footprint are essential enabling tools for PCB decarbonization, not because they yield a single “correct” number, but because they identify dominant hotspots, prevent inappropriate cross-study comparisons, and inform the prioritization of decarbonization strategies summarized in Tables 3 and 4.

### Development of industry standards and regulations

While environmentalism is sometimes viewed as a challenge entangled in legislative complexities and disclosure requirements, forward-thinking companies recognize it as an opportunity rather than an obstacle. Embracing environmental practices yields long-term benefits, replacing negative perceptions with a proactive mindset. Opting for low-emission manufacturing processes or embracing material recycling and recovery not only demonstrates environmental responsibility but also proves financially astute. These choices offer opportunities to reduce costs related to energy consumption, waste treatment, and unnecessary procedures. Companies that prioritize environmentalism position themselves ahead of regulatory changes, staying compliant as legislation tightens. Additionally, such initiatives make companies attractive for environmental, social, and governance (ESG) investments, further emphasizing the strategic advantages of embracing environmental consciousness.

ESG initiatives can help companies reduce costs by implementing energy-efficient practices and recycling e-waste. The companies can target different areas of their organization to implement more sustainable and ethical practices. ESG reporting frameworks are used by companies to disclose data covering business operations, opportunities, and risks related to the ESG aspects of the business. Several studies report associations between ESG performance and financial indicators such as equity returns and downside risk. Siemens serves as a notable example, having set ambitious targets for carbon emissions reduction, increased energy efficiency, and support for social causes. In 2021, Siemens witnessed a remarkable 16% growth in revenue compared to the previous year, surpassing 74 billion USD.^115^ This success underscores the positive correlation between robust ESG commitments and overall financial performance, emphasizing the value of sustainability in the PCB-related business landscape.

Currently, there is a lack of comprehensive regulations mandating companies to implement ESG initiatives. While some countries and regions have introduced rules, the standards vary widely across jurisdictions, causing confusion for companies operating internationally. For instance, in the US, there is no federal mandate requiring companies to disclose their ESG practices, although some states have enacted their own legislation.^116^ This absence of a standardized framework makes it challenging for investors and stakeholders to assess a company’s ESG strategy. Consequently, companies, particularly in regions with minimal regulatory requirements, may be hesitant to invest in sustainable practices, perceiving them as an unnecessary financial burden due to the lack of a uniform regulatory landscape. For PCB supply chains, this regulatory fragmentation directly affects scope 3 emissions accounting, supplier data availability, and the comparability of product carbon footprint disclosures discussed in environmental impact and implementation of life cycle assessment (LCA).

The energy resource management (ERM) systems primarily support scope 2 reductions at the facility level and provide the primary activity data required for facility-to-product allocation approaches used in ISO 14067-based PCB carbon footprint (implementation of life cycle assessment (LCA)). The ERM could be used as guidelines to effectively manage and improve the PCB manufacturing processes.^117^ As sustainability gains prominence in the semiconductor industry, government mandates, and green investment initiatives may offer incentives for sustainable PCBs and electronics. With emissions crackdowns and increasing public awareness of global warming, consumers are actively seeking environmentally responsible companies. To reduce its contribution to global warming, the electronics industry must move away from conventional manufacturing practices, prioritizing the use of low-toxicity, low-emission materials and processes. This transition is becoming increasingly urgent as more companies pledge to meet net-zero targets. Proactively adapting is also strategic, as potential increases in carbon pricing could make renewable energy both more dependable and economically attractive.

Lowering the industry’s carbon footprint remains a substantial challenge, particularly for established manufacturers reluctant to abandon long-standing methods. However, stricter government regulations and heightened consumer expectations worldwide are pressuring producers to take greater responsibility for cutting emissions. Adopting sustainable manufacturing not only delivers environmental gains but also offers economic advantages. Achieving this often requires moving away from legacy, waste-intensive processes and embracing modern techniques and equipment, such as additive manufacturing in place of traditional subtractive methods, to significantly reduce waste and emissions. These governance mechanisms align with the enabling pathways summarized in Table 4.

## Conclusions and future directions

This review shows that, despite advances in recycling and material innovation, no single intervention can deliver net-zero emissions across the PCB supply chain. Life cycle evidence consistently identifies electricity-intensive manufacturing processes, copper-dominated materials, and chemically intensive waste management as the primary environmental hotspots. Near-term emission reductions are most effectively achieved through renewable electricity adoption and energy-efficiency measures, while material substitution, additive manufacturing, and circular strategies mainly address longer-term scope 3 impacts and depend on qualification, traceability, and end-of-life infrastructure. The five proposed strategies should therefore be implemented as an integrated solution, supported by standardized LCA, transparent product carbon footprint, and harmonized governance frameworks to enable credible decarbonization at scale.

### Outlook and future directions

Future decarbonization of the PCB supply chain will be constrained less by the absence of technical options and more by measurement quality, qualification requirements, and infrastructure reality. A priority research need is boundary-consistent, primary-data LCA and product carbon footprint reporting that enables comparisons across board types (e.g., area, layer count, finishes, and yields) and regions (e.g., grid intensity), including uncertainty ranges and transparent allocation choices. On the technology side, electricity decarbonization and process-level energy management remain the most credible near-term levers because manufacturing electricity can dominate cradle-to-gate impacts for multilayer PCBs. Research should therefore couple renewable procurement strategies (e.g., PPAs) with fine-grained energy metering of wet-processing, plating, lamination, drilling, and wastewater treatment to identify the most cost-effective abatement pathways without compromising reliability or yield.

Medium-to long-term mitigation depends on materials and circularity. Promising directions include qualified reductions in copper intensity, verified recycled-content metals, and alternative laminate systems that improve end-of-life separability while meeting IPC-level performance requirements. Circular benefits will only be realized at scale if design-for-recycling is paired with higher collection rates, controlled dismantling, and economically viable routes for the non-metallic fraction. Policy implications include stronger supplier disclosure requirements (product carbon footprint and LCA), harmonized standards for low-carbon procurement, and extended producer responsibility that rewards verifiable, lifecycle-based emissions reductions rather than unverified claims.

### Limitations of the study

This work is a framework-based narrative review with scoping elements rather than a full systematic review or meta-analysis. Evidence quality and comparability are uneven because published PCB LCAs and carbon footprints use different functional units, system boundaries, electricity assumptions, and yield/scrap treatments, while supplier-level scope 3 inventories are often proprietary. Consequently, reported reduction magnitudes are treated as boundary-qualified ranges and case-specific examples rather than pooled averages, and some interventions cannot yet be compared quantitatively due to data gaps.

## Acknowledgments

The authors would like to extend their sincere appreciation to 10.13039/501100006477National Taiwan University for its support under project number 115L7836.

## Author contributions

S.N.: conceptualization, investigation, data curation, visualization, writing – original draft. A.R.: investigation, visualization, writing – review and editing. S.-Y.P.: conceptualization, supervision, writing – review and editing.

## Declaration of interests

The authors declare that they have no known competing financial interests or personal relationships that could have appeared to influence the work reported in this paper.

## Declaration of generative AI and AI-assisted technologies in the writing process

During the preparation of this work the authors used ChatGPT (OpenAI) to assist with language editing and improving clarity. After using this tool, the authors reviewed and edited the content as needed and take full responsibility for the content of the published article.
