# Supplementary material for: Strategies for decarbonizing printed circuit board supply chain
Source: iScience. 2026 Apr 8;29(5):115559. doi: 10.1016/j.isci.2026.115559 (PMC13141769; doi:10.1016/j.isci.2026.115559)
Supplement: Document S1. Figure S1, Tables S1 and S2, and Methods S1–S5 [file mmc1.pdf]

iScience, Volume *29*

Supplemental information

Strategies for decarbonizing printed circuit board supply chain

Suraj Negi, Aishwarya Rani, and Shu-Yuan Pan

Supplementary Information

Strategies for Decarbonizing Printed Circuit Board Supply Chain

Suraj Negi [1], Aishwarya Rani [1], Shu-Yuan Pan [1,2,*]

[1] Department of Bioenvironmental Systems Engineering, College of Bioresources and Agriculture, National Taiwan University, Taipei, 10617, Taiwan ROC

[2] Agricultural Net-Zero Carbon Technology and Management Innovation Research Center, College of Bioresources and Agriculture, National Taiwan University, Taipei City, 10617 Taiwan, ROC.

[*] Corresponding author email: sypan@ntu.edu.tw

Number of Tables: 3

Number of Figures: 1

Number of Pages: 6

Supplementary Methods S1. Review design and scope

This study is a problem-oriented narrative review with scoping elements designed to synthesize heterogeneous evidence on sustainability and decarbonization across the PCB supply chain. The review was guided by two questions: (1) whether the PCB supply chain is environmentally sustainable and (2) whether current practices are sufficient to meet carbon-neutral or net-zero targets. Evidence was structured by lifecycle stage (raw material sourcing, fabrication/assembly, logistics, and end-of-life) and synthesized into five decarbonization pathways: (i) renewable electricity and energy-efficient production, (ii) sustainable materials and design optimization, (iii) circular economy strategies (including recycling/recovery and targeted offsetting), (iv) LCA implementation, and (v) standards and regulatory mechanisms. Due to heterogeneous methods and metrics, synthesis was framework-based and narrative rather than meta-analytic.

Supplementary Methods S2. Evidence identification (targeted database searches + citation chaining)

To strengthen methodological transparency and update recent coverage during revision, targeted keyword searches were conducted in Scopus and complemented by citation chaining and targeted retrieval of standards/policy documents and corporate/industry disclosures. Scopus searches used the following filters: Document type (Article OR Review), Language (English), and Subject areas (Engineering, Environmental Science, Energy, and Materials Science). Two time windows were applied: 2010–2025 (core window) and 2023–2025 (update window).

Six query families were designed to cover the evidence streams required for a full supply-chain synthesis: (i) LCA/carbon footprint quantification, (ii) manufacturing decarbonization and process hotspots, (iii) waste PCB recycling routes and environmental trade-offs, (iv) sustainable/biodegradable substrates and alternative laminates, (v) circular economy/design-for-recycling strategies in electronics/PCBs, and (vi) additive/printed electronics approaches. Counts are not mutually exclusive because records can appear in multiple searches; overlaps were handled during screening.

Supplementary Methods S3. Screening and eligibility

Records were screened at title/abstract level for relevance to printed circuit boards and decarbonization of the PCB supply chain. A specific screening step excluded results where "PCB" referred to polychlorinated biphenyls. Full-text retrieval was prioritized when sources (i) reported quantitative environmental indicators relevant to PCB production or end-of-life, (ii) evaluated interventions relevant to PCB decarbonization, (iii) assessed PCB recycling routes and associated environmental trade-offs, and/or (iv) addressed governance mechanisms applicable to electronics/PCB value chains.

Supplementary Methods S4. Data extraction and synthesis

For LCA/carbon footprint sources, extracted fields included functional unit, system boundary, geographic context/electricity assumptions (when reported), hotspots, and uncertainty/limitations. For manufacturing and recycling studies, extracted fields included process scope, key inputs/outputs, recovery yields, and key

environmental burdens. Evidence was mapped to lifecycle stages and then to the five pathways to support cross-stage comparison and feasibility assessment.

Supplementary Methods S5. Limitations

This review does not claim exhaustive capture of all PCB sustainability literature. Industrial emissions inventories and supplier-level Scope 3 data are frequently proprietary, and published LCA/carbon-footprint studies vary in boundaries, functional units, and data quality; therefore, values are reported with explicit qualifiers and summarized as ranges rather than pooled into a single estimate.

Table S1. Region-wise generation of waste PCBs (WPCBs). Data is sourced from Baldé et al. [S1].

Regions	WPCBs generation (kt)	Formally collected and recycled WPCBs (kt)	Informally collected and recycled WPCBs (kt) (rate in %)	WPCBs total import (kt)	WPCBs Total export (kt)
**Africa**	100	13	87	7	0
**America**	300	132	168	128	65
**Asia**	600	102	498	36	111
**Europe**	300	183	117	184	172
**Oceania**	10	3.1	6.9	4	0
**Worldwide**	**1310**	**433.1**	**876.9**	**359**	**348**

Table S2. Region-wise generation of waste PCBs (WPCBs) per capita. Data is sourced from Baldé et al. [S1].

Regions	Population (millions)*	WPCBs generation (g)	Formally collected and recycled WPCBs (g)	Informally collected and recycled WPCBs (g)	WPCBs total import (g)	WPCBs Total export (g)
**Africa**	1152	86.8	11.3	75.5	6.1	0
**America**	984	304.87	134.1	170.7	130.08	656.1
**Asia**	4445	135	22.9	112.0	8.1	25
**Europe**	740	405	247.3	158.1	248.6	232.4
**Oceania**	42	238	73.8	164.28	65.2	0
**Worldwide**	**7363**	**177.9**	**58.8**	**119.1**	**48.7**	**47.3**

*Population data is taken from Trivedi et al. [S2].

Table S3. Comprehensive comparison of organic PCB and paper-based PCB. (Adapted from Liu et al. [S3]).

Parameters	Organic-based PCB	Paper-based PCB
Substrate	Epoxy resin/glass fiber/inorganic fillers	Commercially available paper
Biodegradability	Non-degradable	Degradable
Flame retardation	Good	Poor to fair
Moisture resistance	Good	Poor to fair
Line spacing (μm)	~50	~100 μm
Line width (μm)	~50	50–100 μm for screen printing
Tensile strength	-	~6 MPa for printing paper
Flexural strength (MPa)	>400	-
Resistivity of conductive material (Ω cm)	~2 × 10−6 for copper foil	~10−5 Ω cm for 50% Ag loading of PU based ECA
Reliability	>85 °C/85RH 1500 h<br>>−40–125 °C per 500 cycles	>85 °C/85RH 1500 h<br>>−40–125 °C per 500 cycles
SMT temperature (°C)	~240	Room temperature
Dielectric constant	~4.5 (1 GHz)	~3 (1 GHz)
Dielectric loss factor	~0.023 (1 GHz)	~0.132 (1 GHz)
Cost (USD/m^2)	90-120	15-30

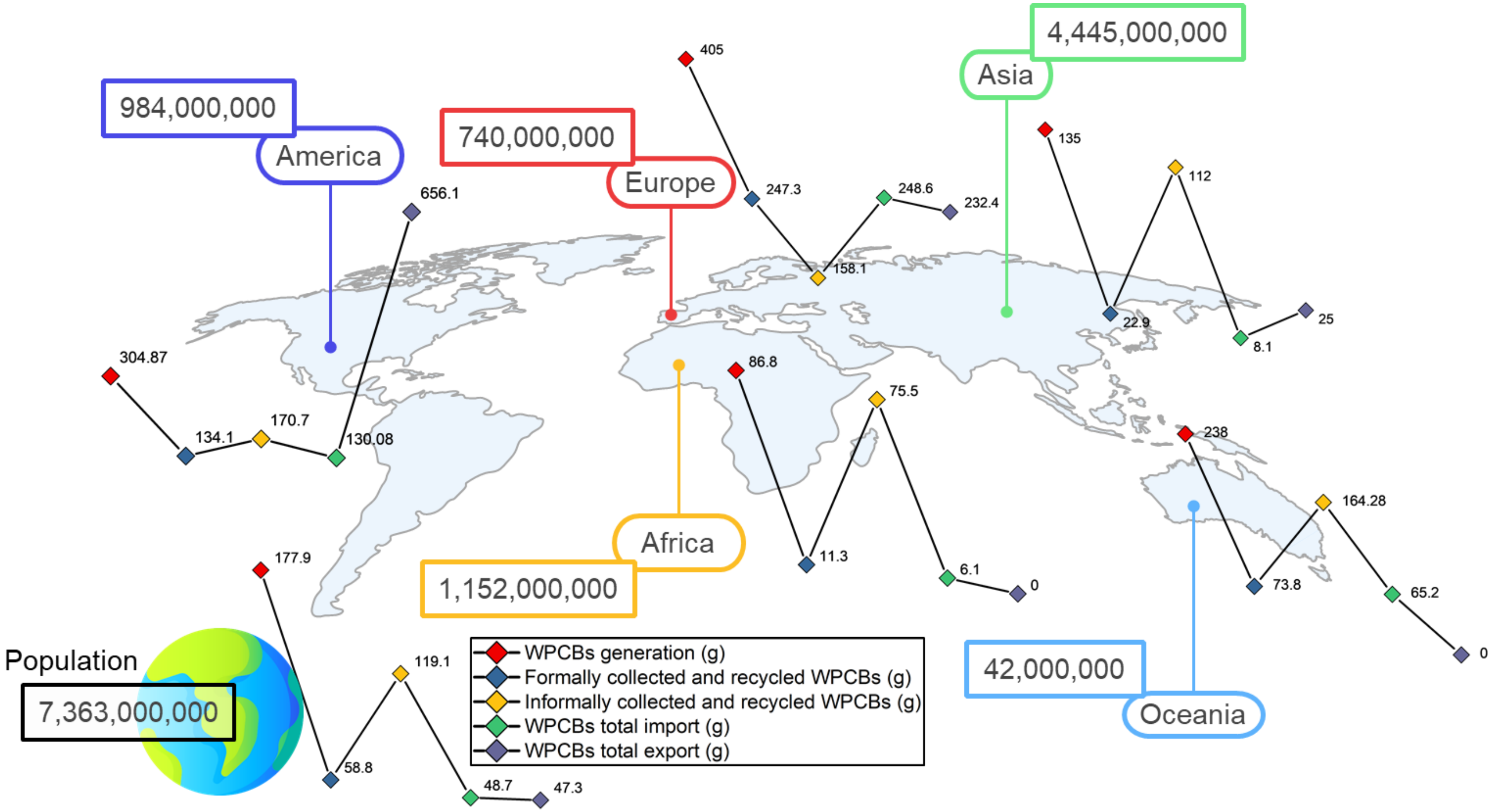

Figure S1. Per capita waste PCBs generation, collection, import, and export for different regions around the world. Data is based on **Table S2.**

References

[S1] C.P. Baldé, E.D.A., V. Luda, O. Deubzer, R. Kuehr (2022). Global Transboundary E-waste Flows Monitor 2022.

[S2] Trivedi, A., Vishwakarma, A., Saawarn, B., Mahanty, B., and Hait, S. (2022). Fungal biotechnology for urban mining of metals from waste printed circuit boards: A review. Journal of Environmental Management 323, 116133.

[S3] Liu, J., Yang, C., Wu, H., Lin, Z., Zhang, Z., Wang, R., Li, B., Kang, F., Shi, L., and Wong, C.P. (2014). Future paper based printed circuit boards for green electronics: fabrication and life cycle assessment. Energy & Environmental Science 7, 3674–3682.
